# A Prediction Model for Deciphering Intratumoral Heterogeneity Derived from the Microglia/Macrophages of Glioma Using Non-Invasive Radiogenomics

**DOI:** 10.3390/brainsci13121667

**Published:** 2023-12-01

**Authors:** Yunyang Zhu, Zhaoming Song, Zhong Wang

**Affiliations:** Department of Neurosurgery, The First Affifiliated Hospital of Soochow University, No. 899, Pinghai Road, Suzhou 215006, China

**Keywords:** glioma, microglia, macrophages, radiomics, immunogenomics

## Abstract

Microglia and macrophages play a major role in glioma immune responses within the glioma microenvironment. We aimed to construct a prognostic prediction model for glioma based on microglia/macrophage-correlated genes. Additionally, we sought to develop a non-invasive radiogenomics approach for risk stratification evaluation. Microglia/macrophage-correlated genes were identified from four single-cell datasets. Hub genes were selected via lasso–Cox regression, and risk scores were calculated. The immunological characteristics of different risk stratifications were assessed, and radiomics models were constructed using corresponding MRI imaging to predict risk stratification. We identified eight hub genes and developed a relevant risk score formula. The risk score emerged as a significant prognostic predictor correlated with immune checkpoints, and a relevant nomogram was drawn. High-risk groups displayed an active microenvironment associated with microglia/macrophages. Furthermore, differences in somatic mutation rates, such as IDH1 missense variant and TP53 missense variant, were observed between high- and low-risk groups. Lastly, a radiogenomics model utilizing five features from magnetic resonance imaging (MRI) T2 fluid-attenuated inversion recovery (Flair) effectively predicted the risk groups under a random forest model. Our findings demonstrate that risk stratification based on microglia/macrophages can effectively predict prognosis and immune functions in glioma. Moreover, we have shown that risk stratification can be non-invasively predicted using an MRI-T2 Flair-based radiogenomics model.

## 1. Introduction

Microglia and macrophages play pivotal roles as immunocytes within the glioma microenvironment [1,2,3,4]. Microglia originate from primitive yolk sac myeloid precursors and derive from primitive myeloid progenitors [1,5]. They represent an ontogenetic population in the mononuclear phagocyte system, establishing residence within the brain during embryogenesis. Conversely, macrophages typically infiltrate the central nervous system in response to pathological cues. Despite their shared phagocytic nature, microglia and macrophages exhibit distinctive behaviors within the tumor microenvironment [6]. Notably, they have been observed to exhibit a low frequency of attacking glioma cells. Instead, their role has been strongly linked to immunosuppression, immune tolerance, tumor proliferation, tumor metastasis, and angiogenesis [2,7,8]. These functions might be due to the factors released by microglia and macrophages, such as stress-inducible protein 1 (STI1), epidermal growth factor (EGF), CSF-1, transforming growth factor-β (TGF-β), and MT1-MMP [9,10,11,12,13,14]. Depletion of microglia has been shown to diminish tumor proliferation and invasiveness [14,15]. The field of immunotherapy has witnessed remarkable progress, particularly in immune vaccine development [16], adoptive cell transfer [17], and immune checkpoint blockade [18,19], though much progress in immunotherapy has been achieved in the treatment of melanoma [20,21], non-small-cell lung cancer [22], and prostate cancer [23]. However, glioma has remained relatively resistant to breakthroughs in immunotherapy. With the development of single-cell sequencing, it has gradually become convenient to study the immune microenvironment for glioma immunotherapy.

Radiomics has become a powerful tool to study the relationship between imaging features and clinical characteristics. It has been applied in predicting IDH mutation [24], prognosis [25], and tumor-infiltrating macrophages [26]. However, the realm of radiogenomics in glioma remains relatively uncharted. Our endeavor seeks to elucidate the potential correlations between radiomic features and immunogenomic risk stratification in the context of glioma.

In the course of our research, we subjected single-cell datasets to dimensionality reduction using Uniform Manifold Approximation and Projection (UMAP) to identify genes correlated with microglia/macrophages. The prognostic and immunogenomic characteristics of different risk stratification groups were studied in detail. A radiomics model was constructed to predict risk stratification groups. It might offer non-invasive and convenient predictions before surgical operations.

## 2. Methods

### 2.1. Dataset Acquisition and Processing

We accessed four single-cell RNA-sequencing (scRNA-seq) datasets for UMAP analysis. CGGA-scRNA-seq data was acquired from the Chinese Glioma Genome Atlas (CGGA), while GSE131928, GSE159416, and GSE193884 datasets were sourced from the Gene Expression Omnibus (GEO) database. The RNA-seq data, along with complete clinical information of 702 glioma patients in The Cancer Genome Atlas Glioblastoma Multiforme and Lower-Grade Glioma (TCGA-GBM/LGG) database, were obtained from UCSC Xena (http://xena.ucsc.edu/ (accessed on 1 January 2023)). From this cohort, 603 patients with information related to survival time, IDH mutation, and 1p19q codeletion were selected for further prognostic study. To validate our findings, we utilized the RNA-seq data and clinical information from the CGGA-325 and CGGA-693 datasets. The datasets included glioblastomas, astrocytomas, oligodendrogliomas, and oligoastrocytomas. The tumors studied in our research encompassed a range of WHO grades, including grades 2, 3, and 4 (the latter grade includes GBM, which is the most aggressive and lethal form of glioma).

The expression levels of hub genes in normal tissue were obtained from the Genotype-Tissue Expression (GTEx) dataset. Batch effect correction was performed using the R package termed “SVA”. Single-nucleotide polymorphism (SNP) data of TCGA-GBM and TCGA-LGG were acquired from UCSC Xena to calculate tumor mutational burdens (TMBs). In addition, MRI data corresponding to TCGA-GBM/LGG patients were obtained from The Cancer Imaging Archive (TCIA). Patients with integral MRI data and clinical information were selected for further radiomics study.

### 2.2. Identification of Microglia/Macrophage-Correlated Clusters by Uniform Manifold Approximation and Projection (UMAP) Algorithm

We harnessed the UMAP algorithm, a non-linear dimensionality reduction technique rooted in manifold learning and topological data analysis, to analyze the single-cell sequencing datasets. The “Seurat” package in R facilitated UMAP application, with data normalization conducted via “LogNormalize” and variable feature selection by employing the “vst” method. Distinct cell clusters were demarcated using established cell markers. Notably, TMEM119 served as a specific marker for microglia, distinguishing them from macrophages. Additional microglia markers included SALL1, P2RY12, and CX3CR1, while macrophage markers encompassed CD68, CD86, CD163, TSPO, and ITGA4. Oligodendrocyte markers were OLIG2, FA2H, UGT8, and CNP, while tumor markers included SOX2, PARP1, CD44, and PTPRZ1. T-cell markers encompassed CD8, CD3, CXCR3, and CCR6.

### 2.3. Construction of a Risk Score Model

We calculated the intersection of genes that correlated with microglia/macrophages in the four scRNA-seq datasets using the “UpSetR” package in R. Then, univariate Cox regression analysis was utilized for the preliminary screening of prognostic genes (*p* < 0.01). Least absolute shrinkage and selection operator (LASSO) regression analysis was applied by “glmnet” package in R for the final selection of hub genes. The LASSO coefficients based on the lambda.1se model were incorporated as coefficients for risk score computation.

$$\mathrm{Risk}\;\mathrm{score}=\sum_{i=1}^{n}\;{\mathrm{coef}}_{i}\times {\mathrm{expression}}_{i}$$

Moreover, we categorized all patients into high-risk and low-risk cohorts using the median risk score. As for the value equal to the median value, we defined them as low-risk groups artificially. We calculated risk scores based on genes with prognostic values. Hence, our risk referred primarily to survival. However, it should not be ignored that behind the differences in survival is heterogeneity itself (e.g., immune microenvironment and main activation pathways in different groups). We used the “survival” package in R to perform a Kaplan–Meier survival analysis, using the log-rank test to compare the survival curves of different groups to determine if there are significant differences between them.

### 2.4. Expression and Enrichment Analysis of Hub Genes

Gene expressions in glioma were obtained from TCGA-GBM/LGG dataset and the expressions in normal tissue were obtained from the GTEx dataset. The expression comparison between glioma and normal tissue was calculated based on transcripts per million reads (TPM) form after log2 transformation. Gene Ontology (GO) and Kyoto Encyclopedia of Genes and Genomes (KEGG) functional enrichment analysis were applied by “clusterprofiler” package. Additionally, we employed Gene Set Enrichment Analysis (GSEA) to elucidate the immunological functions associated with the hub genes, considering the least adjusted *p*-values and q-values.

### 2.5. Correlation among Hub Genes and Immunologic Scores

The stromal scores, immune scores, and ESTIMATE scores were calculated by “estimate” package. The correlogram among hub genes, risk scores, stromal scores, immune scores, ESTIMATE scores, and tumor purity was drawn with “corrgram” package.

### 2.6. Tumor Mutational Burden (TMB)

Somatic mutation data in TCGA-GBM and TCGA-LGG were processed using the “mutect2” principle and obtained from UCSC Xena. The detailed mutation information of each group was visualized using waterfall plots created by the “maftools” R package.

### 2.7. Prognostic Prediction Ability of Risk Score

In TCGA-GBM/LGG, 603 patients with integral information on survival time, age, grade, IDH mutation, and 1p19q codeletion were divided into high-risk groups and low-risk groups according to the median number of risk scores. A total of 313 patients in CGGA-325 and 657 patients in CGGA-693 were used as the validation set. Then, prognostic factors, including risk score, age, grade, IDH mutation, and 1p19q codeletion, were incorporated to construct a nomogram to assess the 1-year, 2-year, 3-year, and 5-year overall survivals (OS) of glioma. TCGA-GBM/LGG dataset was the training set, while CGGA-325 and CGGA-693 were the testing sets. The calibration curves of TCGA-GBM/LGG, CGGA-325, and CGGA-693 were drawn to evaluate the calibration of the model. The concordance index (C-index) was used to evaluate the prediction ability.

### 2.8. Gene Set Variation Analysis (GSVA)

We utilized the gene set variation analysis (GSVA) algorithm, a nonparametric, unsupervised algorithm, to demonstrate the biological function of microglia and macrophages among different risk scores. Pearson correlation analysis was applied to calculate the correlation between the risk score and microglia- and macrophage-related immune processes. A heatmap was created using the “pheatmap” package. Gene lists for immune function were obtained from the AmiGO 2 portal (http://amigo.geneontology.org/amigo (accessed on 1 January 2023)) and http://download.baderlab.org/EM_Genesets/current_release/Human/symbol (accessed on 1 January 2023).

### 2.9. Immune Checkpoints

We calculated the Pearson correlation between the risk score and immune checkpoints in glioma. The correlations among the risk score and immune checkpoint genes such as LAG3, TIM-3, TMIGD2, PD-1, CD200R1, TIGIT, HVEM, CTLA4, and CD47 were visualized using the “circlize” package. To enhance clarity, we displayed the -log10 (*p*-value) in the diagram.

### 2.10. Radiomics Feature Extraction and Processing

MRI images corresponded with TCGA-GBM/LGG patients that were acquired from the Cancer Imaging Archive (TCIA). Currently, T2 fluid-attenuated inversion recovery (Flair) imaging has replaced T1 enhancement imaging in the delineation of tumor borders, especially in LGG. Clear T2 Flair images of 168 glioma patients before the first operation were selected for further study. Format conversion of DICOM to NIfTI, N4 Bias Field Correction, Z-score normalization, and voxel sizes resampling to 1 mm × 1 mm × 1 mm were all realized by “SimpleITK” in Python. After preprocessing, ITK-SNAP was utilized to preliminarily delineate the glioma regions semiautomatically. Then, the regions of interest (ROI) were corrected manually by two experienced neurosurgeons, respectively.

“PyRadiomics” in Python was applied to extract 1439 radiomic features with the following filters. Texture feature filters: first order; shape; gray-level co-occurrence matrix (GLCM); gray-level run-length matrix (GLRLM); gray-level size-zone matrix (GLSZM); gray-level dependence matrix (GLDM); and neighboring gray-tone-difference matrix (NGTDM). Image filters: no image filter (original); wavelet filter; Laplacian of Gaussian (LoG) filter with the kernel size of 1, 2, 3, 4, 5; square of image intensities (Square) filter; square root of the absolute image intensities (SquareRoot) filter; logarithm of the absolute image intensities (Logarithm) filter; exponential filter of the absolute image intensities (Exponential) filter; magnitude of the local gradient of the image (Gradient) filter; and local binary pattern (LBP) filter of 2D and 3D. These features are used to describe different aspects of the image, as well as information such as texture, shape, and intensity in the imaging.

The intraclass correlation coefficients (ICC) of features were estimated by “Pingouin” in Python. Features whose ICC > 0.75 were regarded as reproducible and included for further study.

### 2.11. Risk Stratification Prediction Model of Radiomics Features

All samples were randomly divided into training sets (70%) and testing sets (30%) by “train_test_split” function of “sklearn” in Python. The Z-score normalization of training sets and testing sets was applied by “StandardScaler” function of “sklearn” in Python. Patients were divided into high-risk and low-risk groups according to the median number of immunogenomic risk scores calculated before. *t*-test was applied to estimate the significant difference in features between high-risk group and low-risk group. If the data showed homogeneity of variance (*p* > 0.05) by Levene test, *t*-test would be applied, while Welch *t*-test would be used for the data without homogeneity of variance (*p* < 0.05). The calculation of Levene test, *t*-test, and Welch *t*-test were realized by “scipy” in Python. Features with significant predictive value of risk stratifications were further selected by Lasso algorithm. Random forest (RF) model and Support Vector Machine (SVM) model were used to predict the risk group stratification, respectively, by “sklearn” in Python. Ten-times and ten-fold crossing validation were applied in SVM model, while crossing validation was not necessary for random forest model since the out-of-bag (OOB) scores were true in “sklearn”. The receiver operating characteristic (ROC) curves were performed, and the areas under the curves (AUC) were utilized to assess the accuracy of different models.

## 3. Results

### 3.1. UMAP Clusters of Four scRNA-Seq Datasets

**Figure 1:**
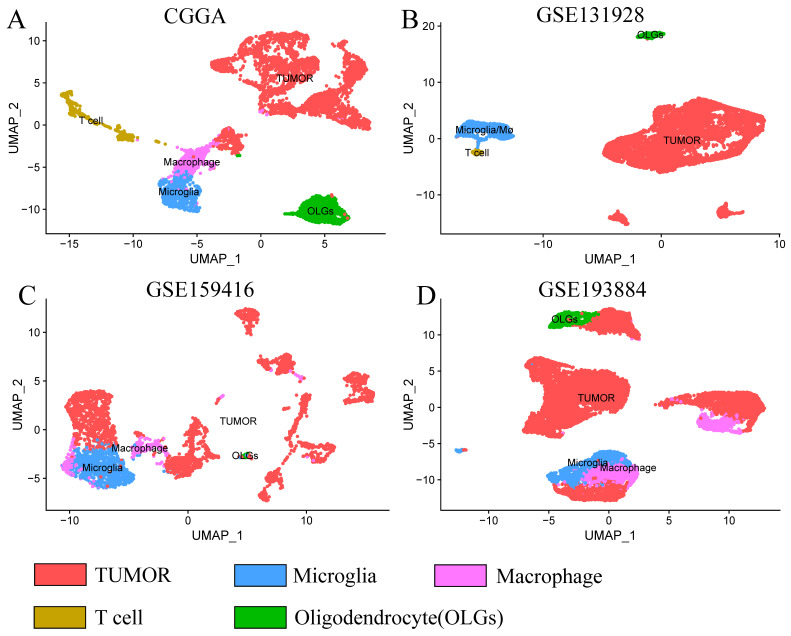
UMAP dimensionally reduced graphic of (**A**) CGGA scRNA−seq dataset, (**B**) GSE131928 scRNA−seq dataset, (**C**) GSE159416 scRNA−seq dataset, and (**D**) GSE193884 scRNA−seq dataset. Red cluster: tumor. Blue cluster: microglia or microglia/macrophages. Lavender cluster: macrophages. Brown cluster: T cell. Green cluster: oligodendrocyte.

**Figure 2:**
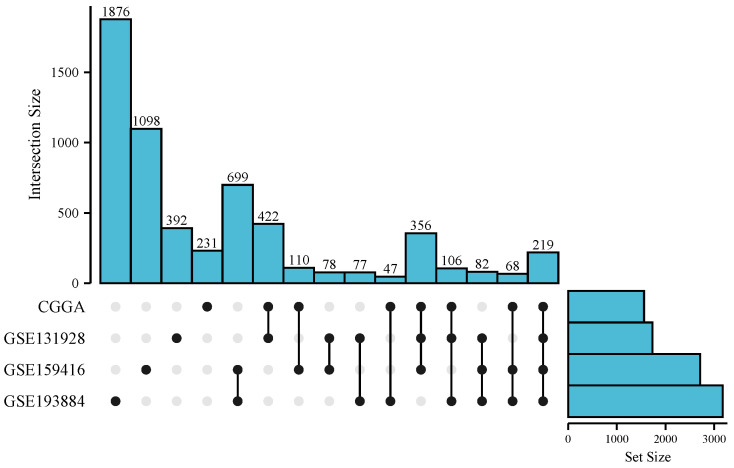
Upset diagram of intersection genes in microglia and macrophage clusters in CGGA, GSE131928, GSE159416, and GSE193884 datasets. The hollow spots represent “without containing” in the dataset, while the solid spots represent “including” in the dataset.

CGGA scRNA-seq dataset was dimensionally reduced into five clusters (Figure 1A), including microglia, macrophages, T cells, oligodendrocytes, and tumors. A total of 1559 correlated genes were included in the microglia cluster and macrophage cluster. GSE131928 scRNA-seq dataset (Figure 1B) was divided into the tumor cluster, microglia/macrophage cluster, oligodendrocyte cluster, and T-cell cluster. Since the distinguishment of microglia and macrophages was difficult, we defined the microglia/macrophage cluster (blue), which contains both of them. A total of 1732 correlated genes were included in the microglia/macrophage cluster. The GSE159416 scRNA-seq dataset (Figure 1C) was dimensionally reduced into the microglia cluster, macrophage cluster, oligodendrocyte cluster, and tumor cluster. A total of 2710 correlated genes were included in the microglia and macrophage cluster. The GSE193884 scRNA-seq dataset (Figure 1D) was divided into the microglia cluster, macrophage cluster, oligodendrocyte cluster, and tumor cluster. A total of 3174 correlated genes were included in the microglia cluster and macrophage cluster. An upset diagram (Figure 2) exhibited the distribution of microglia/macrophage-correlated genes in the four sets. A total of 219 genes included in all four sets were chosen for the next study.

### 3.2. Microglia/Macrophage-Correlated Hub Genes

**Figure 3:**
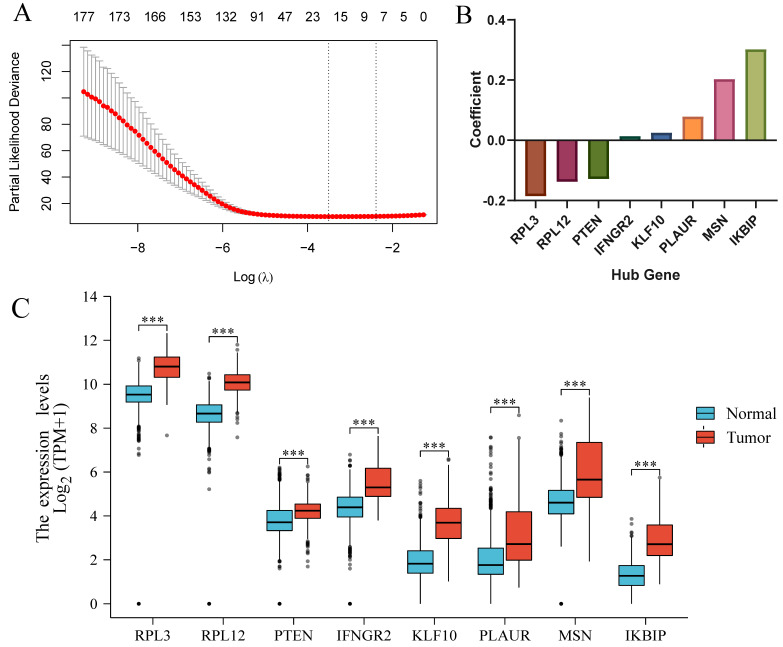
(**A**) The Lasso coefficient spectrum. Red dots represent the validation deviance of different log(lambda) values. (**B**) Histogram of the coefficients among 8 hub genes. (**C**) The expression difference between glioma and normal tissue of 8 hub genes. *** *p* < 0.001.

Univariate Cox regression analysis was utilized to preliminarily choose 178 prognosis-associated genes (*p* < 0.01) among 219 intersection genes. Then, Lasso–Cox regression was implemented to cut out unnecessary coefficients. The Lasso coefficient spectrum (Figure 3A) showed that eight coefficients in lambda.1se would be best and achieve a convenient model. The eight hub genes (Figure 3B) were RPL3 (coef = −0.185786153), RPL12 (coef = −0.138474971), PTEN (coef = −0.128668434), IFNGR2 (coef = 0.013864264), KLF10 (coef = 0.025299618), PLAUR (coef = 0.078538366), MSN (coef = 0.203390786), and IKBIP (coef = 0.302327871). All eight hub genes showed significantly higher expressions in glioma than in normal tissue (Figure 3C). The risk scores were calculated by a summation of expression × coefficient (formula was shown in Section 2).

### 3.3. Functional Enrichment Analysis

**Figure 4:**
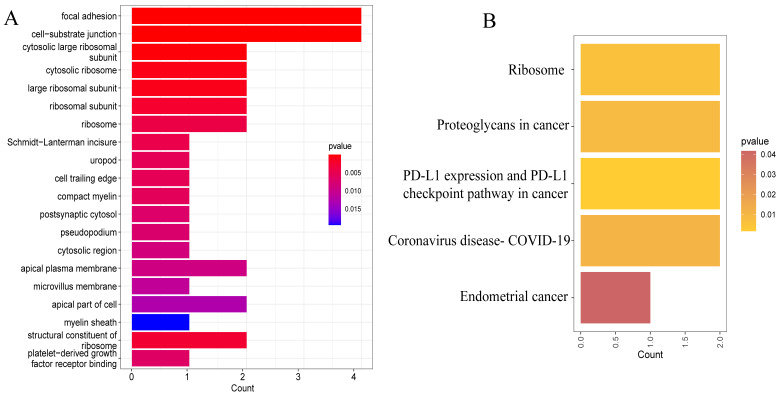
(**A**) Go enrichment analysis of hub genes. (**B**) KEGG enrichment analysis of hub genes.

**Figure 5:**
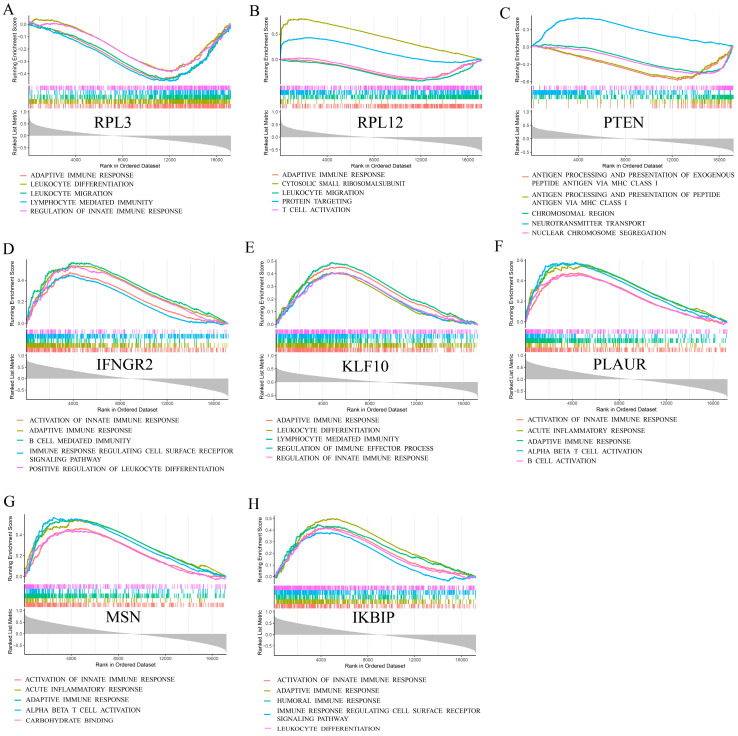
Single-gene GSEA analysis of (**A**) RPL3, (**B**) RPL12, (**C**) PTEN, (**D**) IFNGR2, (**E**) KLF10, (**F**) PLAUR, (**G**) MSN, and (**H**) IKBIP.

Go enrichment analysis (Figure 4A) showed that the biological functions of hub genes were focal adhesion, cell–substrate junction, cytosolic and ribosome, etc. The KEGG enrichment analysis (Figure 4B) revealed that hub genes had the functional pathways of the ribosome, proteoglycans in cancer, PD-L1 expression, and PD-1 checkpoint pathway in cancer, endometrial cancer, and interestingly, coronavirus disease (COVID-19). Single-gene GSEA analysis (Figure 5) of RPL3, RPL12, PTEN, IFNGR2, KLF10, PLAUR, MSN, and IKBIP were calculated, and the top five immunological functions and pathways with the least adjusted *p* values and q values were displayed. Interestingly, RPL3, RPL12, and PTEN, whose coefficients of risk score were negative numbers, had normalized enrichment scores (NES) <−1 in immune processes such as “adaptive immune response”, “leukocyte differentiation”, “leukocyte migration”, “leukocyte mediated immunity”, “innate immune response”, “T cell activation”, “antigen processing, and presentation via MHC class I”. It meant RPL3, RPL12, and PTEN had negative regulation effects on the pathways above. As for IFNGR2, KLF10, PLAUR, MSN, and IKBIP, whose coefficients of risk score were positive numbers, most of the NES values of immunological pathways were also >1. It might indicate that coefficients of risk score had a positive correlation with immunological functions and pathways.

### 3.4. Tumor Mutational Burden (TMB)

**Figure 6:**
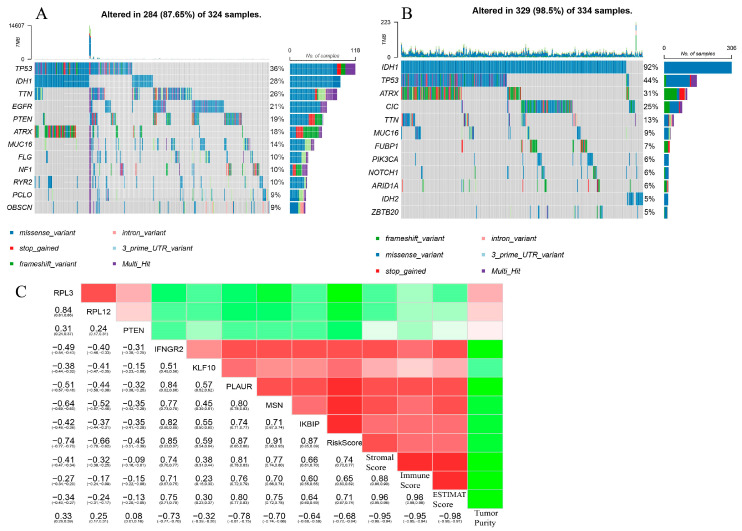
Waterfall plots of tumor mutational burden in (**A**) high-risk group and (**B**) low-risk group. (**C**) Correlogram among hub genes, risk scores, stromal scores, immune scores, ESTIMATE scores, and tumor purity.

According to the median number of risk scores, patients in TCGA-GBM/LGG were divided into a high-risk group and a low-risk group. A total of 324 samples with corresponding mutation information were in the high-risk group, and 334 samples with the information were in the low-risk group. Waterfall plots revealed that TP53 mutation was the main mutation in the high-risk group (Figure 6A), while the IDH1 missense variant was the main mutation in the low-risk group (Figure 6B). The missense variant counted for the majority of TP53, IDH1, TTN, EGFR, PTEN, etc., in the high-risk group and the majority of IDH1, TP53, etc., in the low-risk group. The Frameshift variant counted for the majority of ATRX in both groups, followed by the intron variant. Mutations of IDH1 and ATRX were more frequent in the low-risk group, and mutations of TP53, EGFR, and TTN were more frequent in the high-risk group.

### 3.5. Correlation of Hub Genes and Immunologic Scores

A correlogram (Figure 6C) showed that RPL3, RPL12, and PTEN, whose coefficients of risk score were <0, had negative correlations with risk score, stromal score, immune score, and ESTIMATE score and positive relationships with tumor purity. IFNGR2, KLF10, PLAUR, MSN, and IKBIP, whose coefficients of risk score were >0, had positive correlations with risk score, stromal score, immune score, and ESTIMATE score and negative relationships with tumor purity. The risk score had a positive correlation with stromal score, immune score, and ESTIMATE score.

### 3.6. Prognostic Prediction Ability of Risk Score

**Figure 7:**
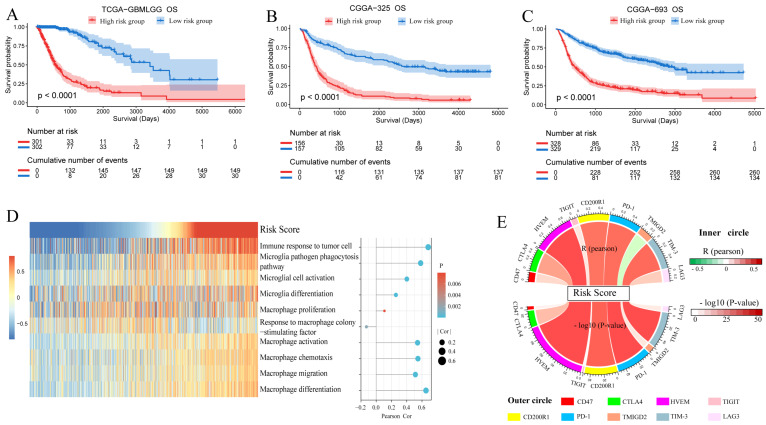
Kaplan−Meier (KM) curves of high-risk group and low-risk group in (**A**) TCGA−GBM/LGG, (**B**) CGGA−325, and (**C**) CGGA−693. (**D**) Heatmap of correlation of risk score and microglia/macrophage immune responses calculated by GSVA. Sphere size of the right part was the absolute value of Pearson R value. Sphere color changed with *p* value. (**E**) Pearson correlation between risk score and immune checkpoints. The color of upper inner part of the circle changed with Pearson R value, and the color of lower inner part changed with Pearson −log10 (*p* value).

A total of 603 patients in TCGA-GBM/LGG with integral clinical information were divided into a high-risk group and a low-risk group according to the median number. Then, 313 patients in CGGA-325, and 657 patients in CGGA-693 were divided by the same rule as applied to validation sets. In the KM curves, the low-risk group showed a significantly better prognosis than the high-risk group in TCGA-GBM/LGG (Figure 7A), CGGA-325 (Figure 7B), and CGGA-693 (Figure 7C). It indicated that the method to dichotomize patients according to risk score had statistical significance in prognostic prediction.

### 3.7. GSVA Indicated the Correlation of Risk Score and Microglia/Macrophage Immune Responses

The heatmap of gene set variation analysis (GSVA) (Figure 7D) revealed that risk score had a remarkable positive correlation with “immune response to tumor cell”, “Microglia pathogen phagocytosis pathway”, “microglial cell activation”, “microglia differentiation”, “macrophage proliferation”, “macrophage activation”, “macrophage chemotaxis”, “macrophage migration”, and “macrophage differentiation”. In addition, the risk score had a negative correlation with “response to macrophage colony-stimulating factor”. The result of GSVA indicated a close correlation between risk score and microglia/macrophage immune responses.

### 3.8. Correlation of Risk Score and Immune Checkpoints

The correlationship among risk score and CD47, CTLA4, HVEM, TIGIT, CD200R1, PD-1, TMIGD2, TIM-3, and LAG3 were revealed by Pearson relevance analysis (Figure 7E). The risk score had positive relevance with checkpoints of CTLA4, HVEM, CD200R1, PD-1, and TIM-3, while it had negative relevance with TMIGD2. Though the *p* value color of TMIGD2 was not deep, as we chose the −log10 (*p*-value) to display it, the actual *p* value of TMIGD2 was 9.61 × 10^−10^. So, the negative relevance of the risk score and TMIGD2 was valid.

### 3.9. Nomogram of Risk Score

**Figure 8:**
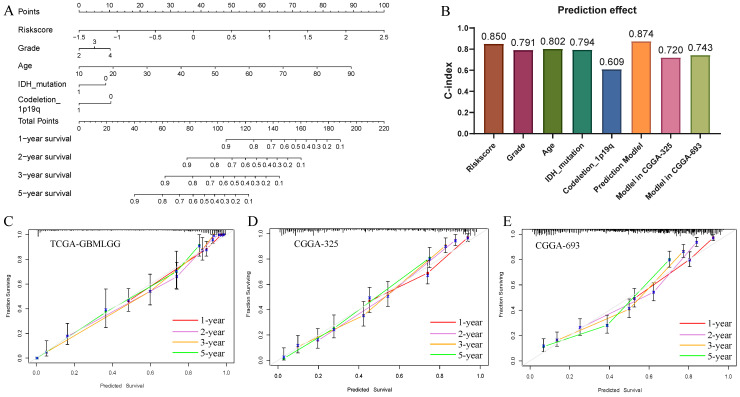
(**A**) A nomogram to assess the 1-year, 2-year, 3-year, and 5-year overall survival (OS) of glioma based on TCGA-GBM/LGG. (**B**) C-index of parameters in nomogram and C-index of the prediction model and validation sets. (**C**) Calibration diagram of TCGA-GBM/LGG. (**D**) Calibration diagram of CGGA-325. (**E**) Calibration diagram of CGGA-693. Red line: 1-year calibration curve. Orchid line: 2-year calibration curve. Orange line: 3-year calibration curve. Limegreen line: 5-year calibration curve.

A nomogram (Figure 8A) containing the parameters of risk score, grade, age, IDH mutation, and 1p19q codeletion was designed to predict the overall survival rate. Integral information with all the parameters of 592 patients in TCGA-GBM/LGG was the training dataset. Integral information of 305 patients in CGGA-325 and 543 patients in CGGA-693 were the testing sets. In the nomogram, risk score and age were the major contributing factors to overall survival. The C-index (Figure 8B) of the risk score was 0.85, indicating a good predictor of prognosis. The C-index of the training set was 0.874. In the testing set, it was 0.72 in CGGA-325 and 0.743 in CGGA-693, showing a good prediction ability. Sterling prediction accuracy was revealed by 1-year, 2-year, 3-year, and 5-year calibration curves in the training set (Figure 8C) and testing sets (Figure 8D,E).

### 3.10. Radiomics Models for Predicting Risk Stratification

**Figure 9:**
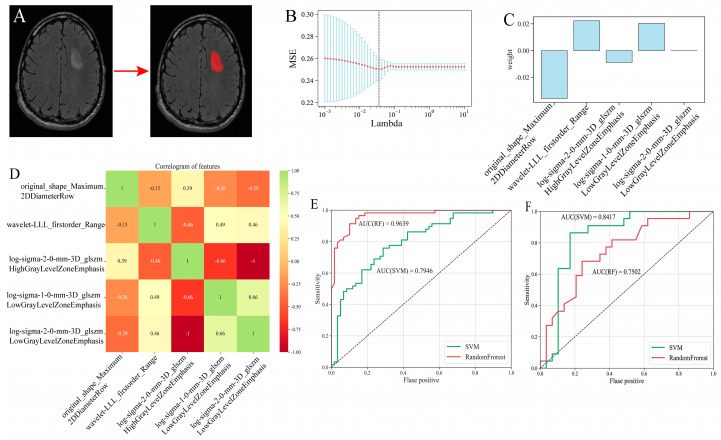
(**A**) Illustration of the ROI delineation. Red area was the ROI region. (**B**) The Lasso coefficient spectrum. Red dots represented the mean squared error of different lambda values. (**C**) Histogram of the lasso coefficients. (**D**) Correlogram of radiomics features. (**E**) ROC curves in training set. (**F**) ROC curves in testing set. Red: random forest. Green: SVM.

T2 flair has shown an unparalleled advantage in the delineation of glioma borders, especially in LGG. So, 168 MR T2 flair images in TCIA corresponding to patients in TCGA-GBM/LGG were selected for the radiomics study. N4 biasField correction, Z-score normalization, and voxel size resampling were carried out before feature extraction. Patients were classified into a high-risk group and a low-risk group by the rules described above. Five radiomics features were finally selected after *T*-test and lasso regression in the training set (Figure 9B). The radiomics features were “original shape Maximum2DDiameterRow” (lasso coef = −0.035783); “wavelet-LLL firstorder Range” (lasso coef = 0.022346); “log-sigma-2-0-mm-3D glszm HighGrayLevelZoneEmphasis” (lasso coef = −0.008832); “log-sigma-1-0-mm-3D glszm LowGrayLevelZoneEmphasis” (lasso coef = 0.020434); and “log-sigma-2-0-mm-3D glszm LowGrayLevelZoneEmphasis” (lasso coef = 0.000006) (Figure 9C). Since features were derived from the same MR imaging, there was some correlation among different features (Figure 9D). The random forest (RF) model and Support Vector Machine (SVM) model were applied to predict the risk group stratification, respectively. C-index has an excellent value in evaluating one single model, while it is not as good as AUC in multiple models. ROC curves were used to evaluate the random forest model and SVM model. In the training set (Figure 9E), the AUC of the random forest model was 0.9639, which was better than 0.7946 in the SVM model. In the testing set (Figure 9F), the AUC of the random forest model was 0.7502, which was worse than 0.8417 in the SVM model. However, the AUC of the SVM model in the testing set was greater than that in the training set. It might result from the lack of sample volume, and the data in the testing set just had a better fit with the SVM model. Finally, the random forest model might be a better model.

## 4. Discussion

In the realm of central nervous system disease, gliomas represent a significant and prevalent form characterized by a daunting prognosis. Despite the integration of immunotherapies, such as adoptive cell transfer and immune checkpoint inhibitors, their efficacy in glioma treatment remains marginal [27]. The main concern of most immunotherapy research eventually returns to T cells and T-cell-related immune pathways [28,29]. Nonetheless, gliomas predominantly feature microglia and macrophages as the key immune cells, marking a deviation from the typical T-cell-focused therapies and signaling a paucity of advancements in glioma immunotherapy.

Our investigation utilized these methodologies to discern microglia/macrophage-associated gene clusters within four multicenter single-cell sequencing datasets. Subsequent prognostic correlation analyses selected hub genes and associated risk scores, while functional enrichment analyses, including GSEA, delineated the immunological functions of these genes. GSVA of the risk score indicated a comprehensive regulatory influence on microglia/macrophages by the hub genes collectively. The risk score demonstrated a robust prognostic prediction capacity, confirmed by a corresponding nomogram. Mutation analysis revealed that IDH1 and ATRX mutations were prevalent within the low-risk cohort, whereas TP53 and EGFR mutations were more common in the high-risk group, aligning with the existing literature that associated the latter with poorer prognoses [30,31]. Moreover, a positive correlation was identified between the risk score and immune checkpoints, namely CTLA4, HVEM, CD200R1, PD-1, and TIM-3, which are implicated in tumor-mediated immunosuppression and evasion, akin to the role of microglia and macrophages in glioma. However, our study design is primarily observational, and therefore, we cannot establish definitive causality based on our results alone. It is worth noting, however, that most of the genes that comprise the risk score have oncogenic effects. In a sense, our findings may be responsible for the development of gliomas.

In the intricate landscape of glioma pathogenesis, the elucidation of risk scores derived from pivotal hub genes—RPL3, RPL12, PTEN, IFNGR2, KLF10, PLAUR, MSN, and IKBIP—emerged as a critical component in understanding the disease’s progression and patient stratification. These genes, through their diverse functions and pathways, have been implicated in the modulation of tumor biology, including proliferation, apoptosis, and the tumor microenvironment’s immune response. RPL3 and RPL12 are part of the ribosomal protein cohort, traditionally viewed as housekeeping genes; however, recent studies suggested their involvement in p53 signaling and cellular stress responses, underpinning their potential roles in tumorigenesis [32,33]. PTEN is a well-documented tumor suppressor gene, and its loss of function mutation is a hallmark of glioma, which would contribute to unregulated PI3K/Akt signaling, promoting oncogenic processes [34]. IFNGR2, the receptor for interferon-gamma, plays a dual role in immune response modulation and is associated with the polarization of microglia and macrophages within the glioma microenvironment, influencing immunogenicity and the efficacy of immunotherapies [35,36]. KLF10, a transcription factor, is implicated in the regulation of TGF-β signaling, a pathway known for its contribution to the immunosuppressive milieu [37,38,39]. PLAUR, or urokinase receptor, is instrumental in extracellular matrix remodeling, a process integral to tumor invasion and angiogenesis. It also affects the recruitment and phenotype of immune cells in the tumor context [40,41]. MSN, an actin-binding protein, has been implicated in the cytoskeletal reorganization associated with cell shape and motility [42], facets central to glioma cell invasion, and interactions with the microenvironment. IKBIP, while less characterized, has been postulated to interact with NF-kB signaling, a pathway with known implications in glioma progression and inflammation, possibly affecting microglial activation states [43].

Radiomics is a non-invasive and convenient method for diagnosis. We extracted image features of MRI T2 flair imagings and tried to construct the model to predict the risk stratification groups. The AUC of the random forest model showed a good prediction effect. However, the AUC of SVM in the testing set was higher than that in the training set. It might be a result of the incompletely consistent distribution, and data in the testing set just better fit the SVM model. More samples were required to reduce this bias. Either way, the random forest model was a better prediction model. 

Our study aimed to find a non-invasive prediction model without craniotomy, and we could not confirm the World Health Organization (WHO) grade and pathological subtypes before the operation. So, although studying gliomas in different grades and pathological subtypes, respectively, would increase the accuracy of prediction models, we still persisted in designing the model in the main category of glioma to imitate the clinical situation without explicit pathological results. We designed the prediction model for the large class of glioma before the identification of the operation and pathological examination. Although it may sacrifice some accuracy, we still believe it is worthwhile.

We used four single-cell RNA datasets to select genes that correlated with microglia and macrophages. Those single-cell datasets had the advantage of cluster analysis, while they lacked clinical data and MRI imaging. Then, we analyzed the prognosis based on TCGA-GBMLGG and CGGA datasets, which had a large number of clinical samples as well as transcriptome gene data. MRI data were obtained from the TCIA dataset. The patients in the TCIA dataset were in one-to-one correspondence with those in TCGA-GBMLGG. Only a few patients lacking appropriate MRI imaging were excluded. Despite the inherent heterogeneity across the datasets analyzed in this study, we employed a compendium of bioinformatics algorithms to mitigate batch effects effectively. A salient limitation, however, was the absence of corroborative in vitro or in vivo experiments to substantiate our bioinformatics predictions. Future experimental research is imperative to elucidate the precise significance and influence of varied cell populations—including microglia, macrophages, and neoplastic cells—as contributory factors in glioma pathogenesis. Additionally, while histological confirmation of specific cell types within the tumor microenvironment is pivotal, our study’s design and methodological framework did not extend to such histopathological evaluations. Typically, the identification of microglia and macrophages in tumor sections necessitates immunohistochemistry (IHC). The unavailability of corresponding IHC-stained sections within TCGA or the single-cell sequencing cohorts precluded this line of investigation in our analysis. Looking forward, it is imperative that our subsequent research incorporates prospective data accrual and meticulous validation studies. Such endeavors will be crucial in ascertaining the generalizability of the identified radiogenomic markers and in establishing their prognostic utility for clinical outcomes in patients with newly diagnosed glioma. More importantly, in a Sankey plot using the CGGA-693 cohort (Figure S1), we found that most patients (71.3%) predicted as being in a high-risk group in our model were finally confirmed as having a high WHO grade (III and IV). It indicated that the parameters of our model, such as hub gene expressions and radiomics features, might have potential correlations with pathological subtypes. Moreover, it revealed that our risk stratification could classify a subset of WHO grade IV patients (18.8%) as low-risk patients (Figure S1). At the same time, 28.7% of WHO grade II patients were also defined as high-risk. This finding held significant implications for guiding treatment selection.

We looked forward to finding some relationships between immunogenomics and radiomics. We also wanted to find a convenient and non-invasive way to assess risk stratification. More imaging cases were required to narrow the bias. Furthermore, deep learning, which could make the imaging–immunogenomics model “smarter”, will be of great necessity in a future study.

## 5. Conclusions

To summarize, our study advanced the understanding of glioma prognostication by integrating the analysis of immune microenvironment heterogeneity through state-of-the-art single-cell sequencing. The identification of hub genes and their associated risk scores unveiled significant prognostic implications. The observed correlation between genetic risk factors and immune checkpoint expression underscored the complexity of glioma immunosuppression mechanisms, potentially guiding future immunotherapy strategies. Moreover, the development of a radiogenomics model enhanced the non-invasive predictive capability, offering a promising tool for risk stratification in clinical settings. Our research paved the way for future explorations that may ultimately lead to improved prognostic tools and personalized therapeutic regimens for glioma patients.

## Data Availability

The datasets used in this article can be acquired from CGGA, TCGA, GEO, GTEx, and TCIA datasets. The gene lists of immune functions were downloaded from the AmiGO 2 portal (http://amigo.geneontology.org/amigo (accessed on 1 January 2023)) and http://download.baderlab.org/EM_Genesets/current_release/Human/symbol (accessed on 1 January 2023).

## Supplementary Materials

The following supporting information can be downloaded at: https://www.mdpi.com/article/10.3390/brainsci13121667/s1, Figure S1: A Sankey plot of risk groups, WHO grades, and histology in the CGGA-693 cohort

## Abbreviation

UMAP (Uniform Manifold Approximation and Projection); STI1 (stress-inducible protein 1); EGF (epidermal growth factor); TGF-β (transforming growth factor-β); scRNA-seq (Single-Cell RNA Sequencing); CGGA (Chinese Glioma Genome Atlas); GEO (Gene Expression Omnibus); TCGA-GBM/LGG (Cancer Genome Atlas–Glioblastoma Multiforme and Lower-grade Glioma); GTEx (Genotype-Tissue Expression); SNP (single-nucleotide polymorphism); TMB (tumor mutational burden); TCIA (The Cancer Imaging Archive); LASSO (least absolute shrinkage and selection operator); TPM (transcripts per million reads); GO (Gene Ontology); KEGG (Kyoto Encyclopedia of Genes and Genomes); GSEA (Gene Set Enrichment Analysis); OS (overall survival); C-index (concordance index); MRI (magnetic resonance imaging); T2 Flair (T2 fluid-attenuated inversion recovery); ROI (regions of interest); GLCM (gray-level co-occurrence matrix); GLRLM (gray-level run-length matrix); GLSZM (gray-level size-zone matrix); GLDM (gray-level dependence matrix); NGTDM (neighboring gray-tone-difference matrix); LoG (Laplacian of Gaussian); SquareRoot (square root of the absolute image intensities); LBP (local binary pattern); RF (random forest); SVM (Support Vector Machine); OOB (out of bag); ROC (receiver operating characteristic); AUC (area under the curve); WHO (World Health Organization).
