# Supplementary material for: A Prediction Model for Deciphering Intratumoral Heterogeneity Derived from the Microglia/Macrophages of Glioma Using Non-Invasive Radiogenomics
Source: Brain Sci. 2023 Dec 1;13(12):1667. doi: 10.3390/brainsci13121667 (PMC10742081; doi:10.3390/brainsci13121667)
Supplement: Supplementary file 1 [file brainsci-13-01667-s001.zip › brainsci-2707658-supplementary.pdf]

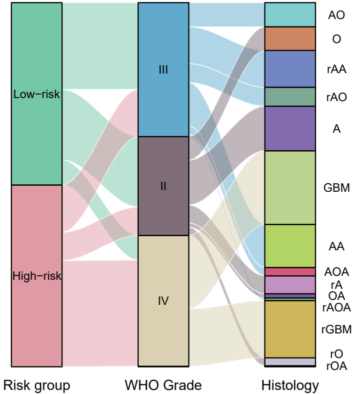

A:astrocytoma O: oligodendroglioma OA: oligoastrocytoma
AA: anaplastic astrocytoma AO: anaplastic oligodendroglioma
AOA: anaplastic oligoastrocytoma r: recurrent
GBM: glioblastoma
